# Meta-analysis of 16S rRNA Microbial Data Identified Distinctive and Predictive Microbiota Dysbiosis in Colorectal Carcinoma Adjacent Tissue

**DOI:** 10.1128/mSystems.00138-20

**Published:** 2020-04-14

**Authors:** Zongchao Mo, Peide Huang, Chao Yang, Sihao Xiao, Guojia Zhang, Fei Ling, Lin Li

**Affiliations:** a School of Biology and Biological Engineering, South China University of Technology, Guangzhou, Guangdong, China; b BGI Genomics, BGI-Shenzhen, Shenzhen, China; University of Massachusetts Medical School

**Keywords:** 16S rRNA, colorectal cancer, adenoma, carcinoma, adjacent tissues, network, supervised learning, enterotype, tissue microbiome, microbial ecology, random forest

## Abstract

As research focusing on the colorectal cancer fecal microbiome using shotgun sequencing continues, increasing evidence has supported correlations between colorectal carcinomas (CRCs) and fecal microbiome dysbiosis. However, large-scale on-site and off-site (surrounding adjacent) tissue microbiome characterization of CRC was underrepresented. Here, considering each taxon as a feature, we demonstrate a machine learning-based method to investigate tissue microbial differences among CRC, colorectal adenoma (CRA), and healthy control groups using 16S rRNA data sets retrieved from 15 studies. A total of 2,099 samples were included and analyzed in case-control comparisons. Multiple methods, including differential abundance analysis, random forest classification, cooccurrence network analysis, and Dirichlet multinomial mixture analysis, were conducted to investigate the microbial signatures. We showed that the dysbiosis of the off-site tissue of colonic cancer was distinctive and predictive. The AUCs (areas under the curve) were 80.7%, 96.0%, and 95.8% for CRC versus healthy control random forest models using stool, tissue, and adjacent tissue samples and 69.9%, 91.5%, and 89.5% for the corresponding CRA models, respectively. We also found that the microbiota ecologies of the surrounding adjacent tissues of CRC and CRA were similar to their on-site counterparts according to network analysis. Furthermore, based on the enterotyping of tissue samples, the cohort-specific microbial signature might be the crux in addressing classification generalization problems. Despite cohort heterogeneity, the dysbiosis of lesion-adjacent tissues might provide us with further perspectives in demonstrating the role of the microbiota in colorectal cancer tumorigenesis.

**IMPORTANCE** Turbulent fecal and tissue microbiome dysbiosis of colorectal carcinoma and adenoma has been identified, and some taxa have been proven to be carcinogenic. However, the microbiomes of surrounding adjacent tissues of colonic cancerous tissues were seldom investigated uniformly on a large scale. Here, we characterize the microbiome signatures and dysbiosis of various colonic cancer sample groups. We found a high correlation between colorectal carcinoma adjacent tissue microbiomes and their on-site counterparts. We also discovered that the microbiome dysbiosis in adjacent tissues could discriminate colorectal carcinomas from healthy controls effectively. These results extend our knowledge on the microbial profile of colorectal cancer tissues and highlight microbiota dysbiosis in the surrounding tissues. They also suggest that microbial feature variations of cancerous lesion-adjacent tissues might help to reveal the microbial etiology of colonic cancer and could ultimately be applied for diagnostic and screening purposes.

## Abstract (precis)

Turbulent fecal and tissue microbiome dysbiosis of colorectal carcinoma and adenoma has been identified, and some taxa have been proven to be carcinogenic. However, the microbiomes of surrounding adjacent tissues of colonic cancerous tissues were seldom investigated uniformly on a large scale. Here, we characterize the microbiome signatures and dysbiosis of various colonic cancer sample groups. We found a high correlation between colorectal carcinoma adjacent tissue microbiomes and their on-site counterparts. We also discovered that the microbiome dysbiosis in adjacent tissues could discriminate colorectal carcinomas from healthy controls effectively. These results extend our knowledge on the microbial profile of colorectal cancer tissues and highlight microbiota dysbiosis in the surrounding tissues. They also suggest that microbial feature variations of cancerous lesion-adjacent tissues might help to reveal the microbial etiology of colonic cancer and could ultimately be applied for diagnostic and screening purposes.

## INTRODUCTION

Colorectal cancer was the third most diagnosed cancer (10.2% of total cases) and the second leading cause of cancer death (9.2% of total cases) worldwide for both sexes in 2018 (1). In 2019, colorectal carcinoma (CRC) was estimated to be the third leading cancer type for both new cases and deaths in the United States, with over 140,000 diagnosed and about 50,000 dead combined in men and women (2). Accurate and early diagnosis is crucial in cancer treatment. Apart from the fecal immunochemical test (FIT), the guaiac-based fecal occult blood test (g-FOBT), and colonoscopy, attempts to investigate the stool microbiota to detect colonic carcinomas and adenomas have been proven to be potentially feasible (3). However, the complete process of how the microbiome interacts with colorectal cancer adjacent tissues is not fully understood.

The development of amplicon and shotgun genome sequencing technologies enabled a better understanding of the relationship between the microbiota and the host. For CRC and colorectal adenoma (CRA), many genera and species have been found to be significantly and consistently enriched or depleted in fecal samples compared with healthy individuals (3, 4). Due to the amplification procedure for DNA preparation, the 16S rRNA protocol outperformed the shotgun method in revealing the tissue microbiome composition (5). By comparing tumor tissue (on-site tissue) samples against the surrounding adjacent tissue samples (off-site tissues), differentially abundant microbial biomarkers were identified (6–8). Moreover, attempts at using the fecal microbiome to detect CRC noninvasively have been put into investigations (5, 9, 10).

However, there may be deficiencies in discriminating disease from the control using the fecal microbiota only. Several meta-analyses have been performed to study microbiome consistency and accuracy based on data sets derived from both amplicon and shotgun genome sequencing (3, 4, 6, 11, 12). Nonetheless, the classification of patients and healthy individuals using fecal microbiota methods was affected by confounders such as ethnic group, diet, and germ line genetic differences of individuals and inherent differences between fecal and tissue microbiomes. Thus, for the fecal microbiota, there is still a long way to transcend the existing screening method (13, 14).

In fact, the tissue microbiome was found to be different from the fecal counterpart (6, 14, 15). Usually, the mucosa or tissue might serve as the perfect environment for specific microbiota to come into effect during tumorigenesis (16, 17). This is especially the case for precancerous lesions, whose fecal microbial dysbiosis is moderate (10). Although several studies had revealed numerous disease-specific species and genera in on-site tissues (6, 18, 19), they have only seldom focused on disease-adjacent ones, which have been found to be difficult to be distinguished from their on-site counterparts using supervised learning methods (6). Moreover, the amplicon sequencing and analysis procedures differ from study to study regarding the hypervariable region, sequencing platform, sequence depth, and bioinformatics pipeline, making it even more challenging to be analyzed uniformly and systemically. Additionally, the results from a previous meta-analysis also showed that fine-scale classification of reads into operational taxonomic units (OTUs) did not help to improve the supervised learning classification performance significantly (12).

In this study, we chose to classify and assign taxonomy annotations to each filtered 16S rRNA gene read using the Kraken2 algorithm (20), making each level of taxa a feature and obtaining feature relative abundances for each sample independently. As a start, we performed a meta-analysis of 15 cohorts of colorectal carcinoma and adenoma samples. After consistent data preprocessing, we obtained feature relative abundances for downstream analyses. First, with the batch effect adjusted, we identified significantly abundant features in different case-control comparisons. Second, after pooling data from different cohorts, we trained random forest (RF) models and evaluated the performance of models in discriminating different sample groups. Importantly, we characterized the pattern of feature relative abundance alterations among sample groups. Third, we computed the correlation coefficient matrices that represented the ecology network and compared the network similarities among groups using the Mantel test. Finally, we investigated cohort heterogeneity and its impact on model classification using the Dirichlet multinomial mixture (DMM) method and cohort-to-cohort (C2C) and leave-one-cohort-out (LOCO) random forest models. Here, we identified distinctive and predictive microbial dysbiosis in the surrounding tissues of on-site colorectal cancer tissues. Importantly, the high similarity between the on-site and off-site colorectal cancer tissue microbiome signatures might provide us with novel perspectives in investigating the tumorigenic role of the microbiota along with the development of colorectal cancer disease.

## RESULTS

### Grouping of colorectal cancer microbiota data sets.

Fifteen 16S rRNA data sets were retrieved from publicly available publications. Patients with CRC and CRA and healthy controls were included in this study, with CRC and CRA collectively called lesions in the following demonstration for convenience. Fecal and on-site and off-site lesion tissue samples were included. Detailed information on the data sets regarding sample size and others is depicted in Table 1.

**TABLE 1:** Sizes of the large-scale 16S rRNA data sets included in this study^a^

Data set	No. of samples									Sequencing platform	Sequencing region
	G-1	G-2	G-3	G-4	G-5	G-6	G-7	G-8	Total		
Zeller	50	38	41				48	48	225	Illumina_MiSeq	V4
Flemer	62	23	69	59	31	2	74	65	385	Illumina_MiSeq	V3_V4
Burns							44	44	88	Illumina_MiSeq	V5_V6
Baxter	172	198	120						490	Illumina_MiSeq	V4
China_GBA				61	52	52	52	52	269	P_454	V1_V4
MAL2							21	23	44	Illumina_MiSeq	V3_V4
MAL1				6			20	21	47	Illumina_MiSeq	V3_V4
Zackular	30	30	30						90	Illumina_MiSeq	V4
Kostic							60	55	115	P_454	V3_V5
PNAS				11	2	2	23	23	61	P_454	V3_V5
Brazil				18				18	36	ION_TORRENT	V4_V5
China_KM							8	8	16	P_454	V1_V2
China_SH				20	31	31			82	Illumina_MiSeq	V3_V4
China_QD	11		10						21	Illumina_HiSeq_2500	V3_V4
China_SHTJ							65	65	130	Illumina_MiSeq	V4

Total	325	289	270	175	116	87	415	422	2,099	NA	NA

a G-1, Normal_Stools (healthy control stool samples); G-2, CRA_Stools (colorectal adenoma stool samples); G-3, CRC_Stools (colorectal carcinoma stool samples); G-4, Normal_Tissue (healthy control colorectal tissue); G-5, CRA_Tissue_Adjacent (colorectal adenoma adjacent tissue); G-6, CRA_Tissue (colorectal adenoma tissue); G-7, CRC_Tissue_Adjacent (colorectal carcinoma adjacent tissue); G-8, CRC_Tissue (colorectal carcinoma tissue); NA, not available.

### Principal-coordinate analysis shows a distinct pattern of clusters.

**FIG 1:**
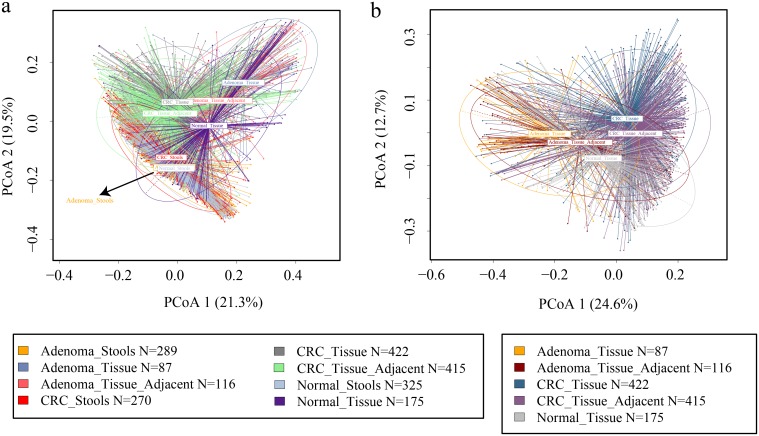
Principal-coordinate analysis (PCoA) in viewing groups of samples. (a) The beta diversity based on the Bray-Curtis metric was used to perform PCoAs. The first two principal coordinates were graphed to visualize the sample group relationships. (b) A similar procedure was applied to CRA and CRC tissue-associated samples to demonstrate particular sample relationships. Each ellipse in different colors represents 95% of the inertia of the corresponding group. Group tags that are not legible due to overlap are specified using dark arrows.

In our ordination analysis based on Bray-Curtis dissimilarity, extensive variations concerning sample groups were observed (Fig. 1a). For the tissue microbiota, principal-coordinate analysis (PCoA) showed distinguishing distributions between lesion, lesion-adjacent tissue, and normal control groups (Fig. 1b; see also Fig. S2B and C in the supplemental material), while for the adenoma stool group, the distribution was not significantly different from that of the normal stool group (*P* = 0.211 for Adenoma_Stools-VS-Normal_Stools as determined by analysis of molecular variance [AMOVA]) (Fig. S2D). Visible separation among cancerous lesion tissues, lesion-adjacent tissues, and healthy colon tissues indicated that the underlying ecological discrepancy among them could be distinguishable.

10.1128/mSystems.00138-20.1 FIG S1 Read profile visualization. (Left) Box plot with orange dots representing the read classification rate of Kraken2. (Right) Box plot with pine green dots representing the log_10_-transformed number of reads in each cohort after Trimmomatic filtering and Kraken2 taxonomy assignment. Download FIG S1, PDF file, 0.9 MB. Copyright © 2020 Mo et al. 2020 Mo et al. This content is distributed under the terms of the Creative Commons Attribution 4.0 International license.

10.1128/mSystems.00138-20.2 FIG S2 PCoA in viewing groups of samples. (A) PCoA plot visualizing the cohort factors across samples. (B) PCoA plot reflecting the distribution of adenoma-associated tissue groups. (C) PCoA plot reflecting the distribution of carcinoma-associated tissue groups. (D) PCoA plot reflecting the distribution of lesion stool groups. In panels B to D, healthy control groups were added for comparison. Download FIG S2, PDF file, 2.0 MB. Copyright © 2020 Mo et al. 2020 Mo et al. This content is distributed under the terms of the Creative Commons Attribution 4.0 International license.

### Differential abundance analysis identifies significantly enriched and depleted features in various case-control strategies.

**FIG 2:**
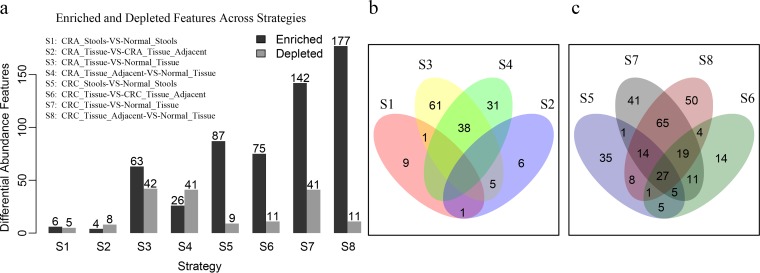
Differential abundance analyses identify enriched and depleted features in different strategies. (a) Differential abundance analysis was conducted by using the DEseq2 software algorithm for each strategy, with an absolute log_2_-fold change above 1 and an adjusted *P* value of less than 0.05 considered enriched or depleted. The cohort was added as a factor to adjust the batch effect. The operator “-VS-” was organized in a form such that the former was compared against the latter, with the enrichment and depletion concepts based on the former. (b and c) Venn plots showing the overlap of the features among different strategies for adenoma- and carcinoma-associated diseases.

We deployed eight differential abundance analysis (DAA) strategies, S1 (CRA_Stools-VS-Normal_Stool) (*n* = 614), S2 (CRA_Tissue-VS-CRA_Tissue_Adjacent) (*n* = 203), S3 (CRA_Tissue-VS-Normal_Tissue) (*n* = 262), S4 (CRA_Tissue_Adjacent-VS-Normal_Tissue) (*n* = 291), S5 (CRC_Stool-VS-Normal_Stool) (*n* = 595), S6 (CRC_Tissue-VS-CRC_Tissue_Adjacent) (*n* = 837), S7 (CRC_Tissue-VS-Normal_Tissue) (*n* = 597), and S8 (CRC_Tissue_Adjacent-VS-Normal_Tissue) (*n* =590) (where VS stands for “versus”), to investigate the potential microbiota differences. In our CRA_Stools-VS-Normal_Stools strategy, only a few features were found to be significantly enriched or depleted (Fig. 2a). Consistent with data from previous studies, compared to healthy stool samples, Porphyromonas endodontalis (false discovery rate [FDR]-adjusted *P* value of 1.28e−20), *Fusobacterium* (FDR-adjusted *P* value of 1.07e−36), Prevotella intermedia (FDR-adjusted *P* value of 9.23e−7), and *Parvimonas* (FDR-adjusted *P* value of 7.87e−65) were found to be significantly enriched in CRC stool samples (Table S2) (4, 12), while only 9 features were founded to be significantly depleted (Fig. 2a). There were 63 and 142 enriched and 42 and 41 depleted features in the CRA_Tissue and CRC_Tissue groups compared to the normal tissues. However, 26 enriched and 41 depleted features could also be observed by strategy S4, while for S8, the numbers were 177 and 11, respectively. This led us to find 38 and 125 overlapping features between strategies S3 and S4 and between S7 and S8, respectively (Fig. 2b and c). For the consideration that a large number of overlaps might simply be due to the fact that the adjacent cancer tissues and cancer tissues were paired and derived from the same individual, we conducted differential abundance analysis by keeping 336 pairs of carcinoma patients and 83 pairs of adenoma patients separated (Fig. S3A). We still found many overlapping features between strategies S3 and S4 and between S7 and S8 (Fig. S3B and C). All results suggested that the microbiota shared between lesions and their adjacent tissues could be undervalued. Detailed results for DAA features in each strategy are shown in Table S2.

10.1128/mSystems.00138-20.3 FIG S3 Illustration of the influence of sample pairing information in overlaps between strategies S4 and S5 and strategies S7 and S8. (A) Schematic diagram of DEseq2 analysis with pairing information considered. (B and C) Overlapping features between strategies S4 and S5 for adenoma and strategies S7 and S8 for carcinoma. Download FIG S3, PDF file, 0.2 MB. Copyright © 2020 Mo et al. 2020 Mo et al. This content is distributed under the terms of the Creative Commons Attribution 4.0 International license.

10.1128/mSystems.00138-20.7 TABLE S1 Detailed information on DNA extraction methods, colonic preparation, sample collection, sequencing platform, and sequencing region of cohorts retrieved. The blank fields indicate that the information was not available. Download Table S1, XLS file, 0.1 MB. Copyright © 2020 Mo et al. 2020 Mo et al. This content is distributed under the terms of the Creative Commons Attribution 4.0 International license.

10.1128/mSystems.00138-20.8 TABLE S2 Differentially abundant features detected by DEseq2 for eight strategies. For strategy S4 and S8, the concept of up and down was described based on the normal tissue group. Download Table S2, XLS file, 0.2 MB. Copyright © 2020 Mo et al. 2020 Mo et al. This content is distributed under the terms of the Creative Commons Attribution 4.0 International license.

### Microbiome CRC and CRA classification models.

To learn about the extent to which the microbial components were different among sample groups and the capacity of the use of microbial information to discriminate colorectal neoplastic diseases, we established random forest (RF) classifiers for all eight strategies by pooling samples (pooling RF model). We decoded the sequencing platform and 16S rRNA hypervariable region information as binary features and estimated their effects on our RF models. Because other factors such as colon preparation method, sample collection, and DNA preparation before sequencing were highly heterogeneous and difficult to standardize, we included cohort factor as a binary feature to assess the impact of cohort heterogeneity. Features maximizing the AUC (area under the curve) value or making the AUC value reach a plateau were selected (Fig. S4A). Additionally, we conducted the same pooling RF analysis without adding these binary features and achieved a similar performance (Fig. S3B). The importance of binary features was relatively low, except for strategy S3 (Fig. S3C).

10.1128/mSystems.00138-20.4 FIG S4 Feature number decisions in 10-fold cross-validation random forest procedures. The AUC of each strategy was calculated and is depicted with the feature number progressively increased from 2 to 100. The applicable threshold of feature numbers was decided empirically when the AUC reached a plateau or a global optimal value. (A and B) Distribution of AUC values of random forest procedures with and without cohort, sequencing platform, and hypervariable region information added. (C) Rank of binary features such as cohort, sequencing platform, and hypervariable region in optimized random forest models. Download FIG S4, PDF file, 1.0 MB. Copyright © 2020 Mo et al. 2020 Mo et al. This content is distributed under the terms of the Creative Commons Attribution 4.0 International license.

**FIG 3:**
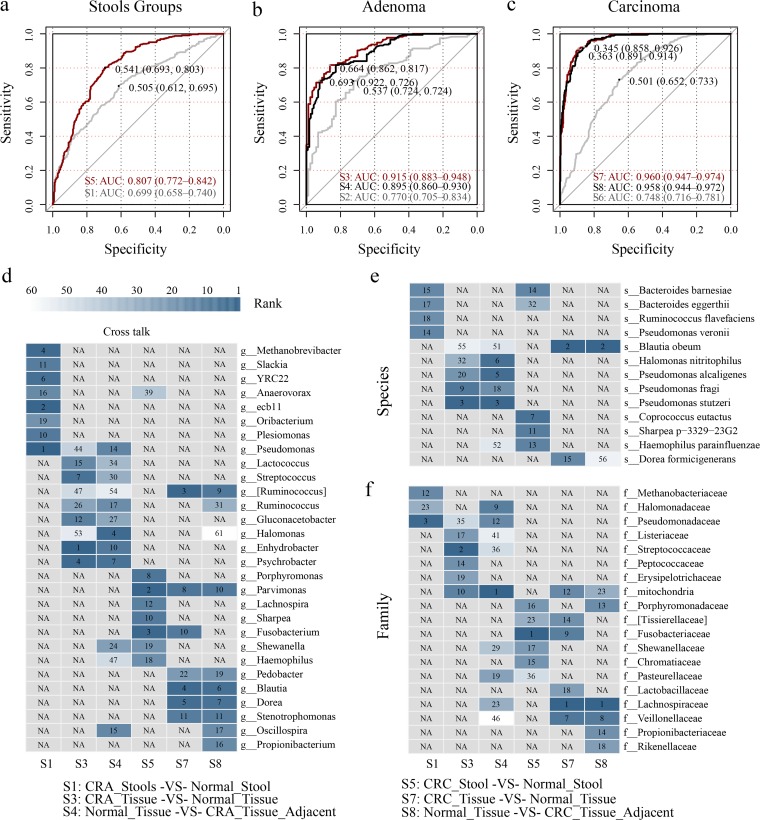
Pooling random forest models and feature cross talk. (a) Random forest models in predicting adenoma and carcinoma using stool samples with 10-fold cross-validation. (b and c) Pooling random forest models in predicting adenoma and carcinoma using tissue samples. (d to f) Genus-, species-, and family-level features’ ranks were obtained according to the random forest model’s MDIA and plotted in a heat map, with dark steel blue for the highest rank, white for the lowest rank, and gray for those that were unavailable. All the strategy codes were carried on as described above. All specificity and sensitivity thresholds were decided by the Youden index. NA, not available.

When using all the CRC and control stool samples to train and predict CRC with 10-fold cross-validation, the AUC was 80.7% (95% confidence interval [CI], 77.2% to 84.2%) in our model containing 61 features. The performance of our model was similar to that in recently reported research based on shotgun sequencing data measured by the AUC (3, 4, 11). Interestingly, when training and predicting CRA using stool samples, the best AUC was 69.9% (95% CI, 65.8% to 74.0%), which showed no deficiency compared with previous studies using shotgun sequencing (Fig. 3a) (3, 4).

In our CRA_Tissue-VS-Control_Tissue and CRC_Tissue-VS-Control_Tissue random forest models, the AUC values were 91.5% (95% CI, 88.3% to 94.8%) and 96.0% (95% CI, 94.7% to 97.4%), respectively (Fig. 3b and c). The top-ranked features of the former model were *Enhydrobacter*, *Streptococcaceae*, Pseudomonas stutzeri, *Psychrobacter*, *Streptococcus*, *Peptococcaceae*, and *Gluconacetobacter*, while the features Blautia obeum, *Dorea*, *Ruminococcus*, *Lachnospiraceae*, *Blautia*, mitochondria, *Parvimonas*, and *Fusobacterium* showed high importance scores in the latter model.

The CRA_Tissue_Adjacent-VS-Normal_Tissue model gave an outstanding performance, with an AUC value of 89.5% (95% CI, 86.0% to 93.0%) (Fig. 3b), and a better performance can be observed for the CRC_Tissue_Adjacent-VS-Normal_Tissue model (AUC = 95.8%; 95% CI, 94.4% to 97.2%) (Fig. 3c). In the CRC_Tissue-VS-CRC_Tissue_Adjacent and CRA_Tissue-VS-CRA_Tissue_Adjacent models, the AUC values were 77.0% (95% CI, 70.5% to 83.4%) and 74.8% (95% CI, 71.6% to 78.1%), respectively. A similar trend was reported in a previous study (6). Selected features for each strategy are shown in Table S3. There was no significant difference between the Normal_Tissue-VS-CRC_Tissue and CRC_Tissue_Adjacent-VS-Normal_Tissue strategy receiver operating characteristic (ROC) curves (*P* = 0.824 by DeLong’s test) for either adenoma phenotype (*P* = 0.402 by DeLong’s test). However, the use of the adjacent tissue microbiome was significantly better than the use of the stool microbiome for both carcinoma (*P* = 2.797e−14 between strategies S5 and S8 by DeLong’s test) and adenoma (*P* = 1.955e−12 between strategies S1 and S4 by DeLong’s test) detection.

10.1128/mSystems.00138-20.9 TABLE S3 Feature importance of optimized pooling random forest models. All importance is represented as the MDIA. The standard deviation was derived from 10-fold cross-validation. Download Table S3, XLS file, 0.1 MB. Copyright © 2020 Mo et al. 2020 Mo et al. This content is distributed under the terms of the Creative Commons Attribution 4.0 International license.

### Microbiota cross talk is widespread between models based on lesion tissues and lesion-adjacent tissues.

Although both carcinoma and adenoma diseases could be efficaciously predicted or discriminated from healthy controls using stool, on-site tissue, and lesion-adjacent tissue samples in our pooling random forest models to different degrees, we found that some features consistently ranked highly in all or most of the strategies in discriminating specific lesions. For instance, *Parvimonas* ranked highly in all three strategies in predicting CRC, while *Ruminococcus*, *Stenotrophomonas*, *Blautia*, *Dorea*, and some other genera were shared by strategies S7 and S8, which used carcinoma on-site tissues and the surrounding adjacent tissues. As for the prediction of adenoma, *Enhydrobacter*, *Psychrobacter*, *Lactococcus*, and *Pseudomonas* were those that cross talked actively between strategies S3 (CRA_Tissue-VS-Normal_Tissue) and S4 (CRA_Tissue_Adjacent-VS-Normal_Tissue) (Fig. 3d). Additionally, some species-level and family-level features also harbored high importance as measured by the mean decrease in accuracy (MDIA) rank (Fig. 3e and f).

### Parallel and divergent feature enrichment and depletion patterns might help reveal microbial distribution across disease development.

**FIG 4:**
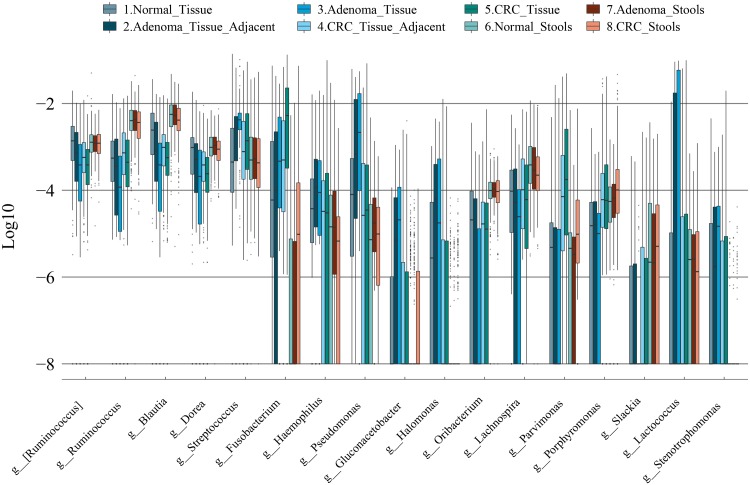
Consistent and divergent genus-level feature profiles along with the temporal order. Box plots of highly cross-talking genus-level features showing consistent and divergent profiles across different groups were arranged in theoretical disease development time series for both tissue and stool samples. A relative abundance of zero was set to 10e−9 to avoid an infinite number.

Adenoma, the precursor of the majority of CRCs (21), was less malignant than CRC and harbored microbiome profiles different from those of CRC (10). We arranged our feature relative abundance profiles in the assumptive Normal_Tissue, CRA_Tissue_Adjacent, CRA_Tissue, CRC_Tissue_Adjacent, and CRC_Tissue temporal order for our presentation. In our pooling analyses, microbial feature relative abundance evidence supported the continuous alteration patterns of the microbiota in CRAs and CRCs. For instance, some taxa, like *Fusobacterium*, showed a gradual accumulation, while others, like *Ruminococcus*, *Blautia*, and *Dorea*, showed progressive depletions along the temporal sequence (Fig. 4). However, we also found some features that were enriched in adenoma tissues and the corresponding adjacent tissues but not prevalent in carcinoma-associated tissues, such as *Streptococcus*, *Haemophilus*, *Pseudomonas*, *Gluconacetobacter*, *Halomonas*, and *Lactococcus*. Similarly, compared with healthy tissues, *Parvimonas* and *Porphyromonas* were enriched in CRC tissues and CRC adjacent tissues but not dominant or even depleted in adenoma-related tissue groups (Fig. 4). These results indicated that the dynamic community change might reflect the succession of different microbiota during colonic cancer development.

### Coabundance analysis identifies highly correlated microbial networks between lesions and corresponding adjacent tissues.

**FIG 5:**
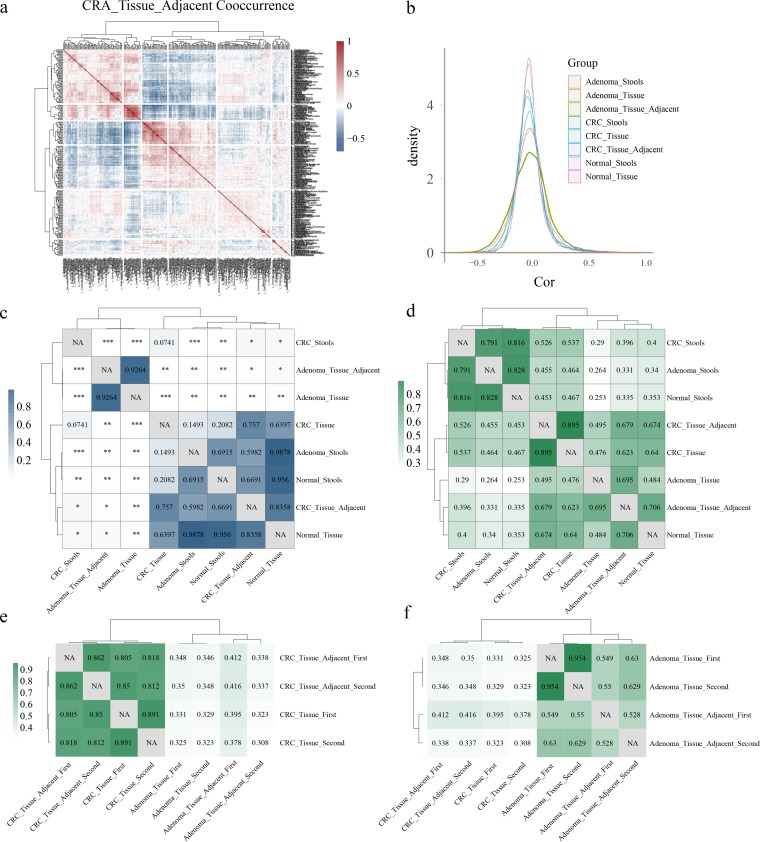
Taxon-taxon correlation coefficient profiles. (a) The coabundance group in adenoma adjacent tissue samples based on the SparCC correlation coefficient was hierarchically clustered to show cooccurrence features. (b) Kernel density estimates of correlation coefficients derived from SparCC for the eight strategies. (c) *P* values of correlation intensions were calculated between different types of samples using correlation coefficient intensity data by the Wilcoxon rank sum test. ***, *P* < 0.001; **, 0.001 < *P* < 0.01; *, 0.01 < *P* < 0.05. (d) Correlation of networks among different samples. The Mantel test was performed between different types of samples using the correlation coefficient matrix to identify the Mantel *r* statistic representing the extent to which the two matrices were correlated. All the correlations presented were significant, and *P* values are not shown (*P* < 0.001). (e and f) The Mantel test was performed with paired lesion tissues and lesion-adjacent tissues separated.

We inferred the taxon-taxon correlation coefficient matrix for each type of sample with all filtered features using SparCC (22) software. After hierarchical clustering, features harboring similar coefficient profiles gathered together as clusters, which is referred to as the coabundance group here (Fig. 5a) (8). First, adenoma adjacent tissues harbored the largest number of positive and negative correlations above the absolute threshold of 0.3 (Fig. 5a and b). Second, after computing the kernel density estimation of the correlation coefficient intensity for each type of sample, we found that the higher coefficients were more prevalent in tissues than in stool samples (Fig. 5b). Consistent with the results of a previous study (23), we observed more correlations higher than 0.3 (*n* = 975) than those lower than −0.3 (*n* = 191) in the CRC_Stools groups. The same result could also be seen when all eight sample groups were aggregated together. To determine whether the differences in interaction strength (absolute value of the correlation coefficient) among sample groups were significant, *P* values were calculated using the Wilcoxon rank sum test. Neither the overall interaction strength between CRA_Tissue_Adjacent and CRA_Tissue (*P* = 0.926) nor that between CRC_Tissue_Adjacent and CRC_Tissue (*P* = 0.757) was significantly different (Fig. 5c).

Using the Mantel test, correlations between each of two networks were calculated. The stool samples showed high correlations, with correlation coefficients between CRC_Stools and Normal_Stools, Adenoma_Stools and Normal_Stools, and CRC_Stools and Adenoma_Stools being 0.816, 0.828, and 0.791 (all *P* < 0.001), respectively. Surprisingly, the correlations between CRC_Tissue and Normal_Tissue, Adenoma_Tissue and Normal_Tissue, and CRC_Tissue and Adenoma_Tissue were 0.64, 0.484, and 0.476 (all *P* < 0.001), respectively (Fig. 5d). This result suggested that microbial networks were more divergent in tissue groups than in stool groups, which was consistent with our random forest discriminating results (Fig. 3a to c). Among the correlation values between carcinoma tissues and other groups, the highest one was 0.895 (between CRC_Tissue and CRC_Tissue_Adjacent), while for the adenoma tissues, the highest value was 0.695 (between Adenoma_Tissue and Adenoma_Tissue_Adjacent). After separating paired samples from the same individuals, as depicted in Fig. S3A, we still observed high correlations between the CRC_Tissue and CRC_Tissue_Adjacent and between the Adenoma_Tissue and Adenoma_Tissue_Adjacent groups (Fig. 5e and f).

### Metacommunity partition reveals cohort-specific patterns of microbial ecology in tissues.

When using the DMM method to decide the optimized number of metacommunities or partitions in tissue samples, including normal controls, carcinoma tissues, adenoma tissues, and their corresponding surrounding adjacent ones, the best partition number was 9 under the Bayesian information criterion (BIC) estimate (Fig. S5A). The sample number and percent distribution in each metacommunity are depicted in Fig. S5B and C.

10.1128/mSystems.00138-20.5 FIG S5 Enterotypes of tissue samples and characteristics. (A) DMM method in deciding the optimal cluster number using BIC estimates. (B and C) Compositions of tissue sample types in different metacommunities, represented by sample numbers and sample percentages. (D and E) Alpha diversities of corresponding metacommunities characterized by chao1 and Simpson metrics based on the feature relative abundance. (F) Selected features’ relative abundances across metacommunities. Feature relative abundances were log_10_ transformed to avoid infinite numbers. Download FIG S5, PDF file, 1.7 MB. Copyright © 2020 Mo et al. 2020 Mo et al. This content is distributed under the terms of the Creative Commons Attribution 4.0 International license.

Harboring the lowest percentage (*n* = 11; 7.6%) of carcinoma on-site tissues, metacommunity A mainly contained benign samples (*n* = 133; 92.4%) and showed low chao1 alpha diversity. Features such as *Fusobacterium* (24) and *Parvimonas*, which were thought to be highly correlated with carcinogenesis, showed low relative abundances (Fig. S5F). Containing the largest number of carcinoma adjacent samples (*n* = 91; 52.9%), metacommunity B showed a sparse microbial ecology, represented by low chao1 alpha diversity as well (Fig. S5D). Consisting of the second-highest percentage of benign samples (*n* = 74; 91.4%), metacommunity C contained no normal tissue and harbored the highest relative abundances of *Lactococcus* and *Pseudomonas*. Metacommunity D, in which 88.5% of the samples were carcinoma related, showed the highest average chao1 (Fig. S5D) and relatively high Simpson alpha diversity (Fig. S5E) values. In metacommunity E, 23.7% (*n* = 40) of the samples were from the normal tissue group, with the rest of the samples being carcinoma related. In metacommunity F, which showed the highest average Simpson alpha diversity value, 91.1% of samples were carcinoma related.

**FIG 6:**
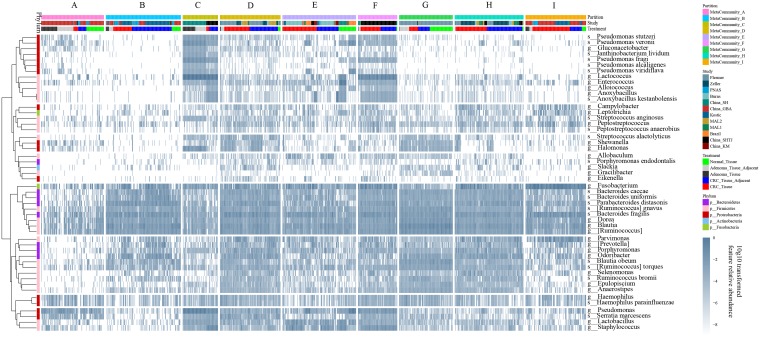
The DMM method identifies different enterotypes representing the microbiome profile. All 657 filtered features of 1,215 tissue samples, including normal samples, adenomas and carcinomas, and the corresponding adjacent tissues, were subjected to the DMM model, resulting in 9 metacommunities. Genus- and species-level taxa that overlapped in DEseq2 and pooling random forest models across 8 strategies were hierarchically clustered using the Pearson correlation and are presented in rows. The feature relative abundance was log_10_ transformed, with an abundance of zero set to 10e−9 to avoid infinite numbers.

Similar to metacommunity C, metacommunity F showed high abundances of *Lactococcus* and *Pseudomonas*. Both metacommunities C and F were mainly from China (China_SH [China, Shanghai] [38] and China_SHTJ [China, Shanghai Tongji] [40]) and prevalently harbored adenoma-related and carcinoma-related samples, respectively. In metacommunity G, we found the largest number of normal control tissue samples (*n* = 53). Enrichments of microbiota taxa like *Dorea*, *Ruminococcus*, and *Blautia* and depletions of *Fusobacterium* and *Parvimonas* (Fig. S5F) were also identified. Collectively, both metacommunities A and G harbored a large number of healthy tissue samples and were represented by low and high chao1 diversity values, respectively. Interestingly, features like *Ruminococcus*, *Blautia*, and *Dorea* showed low abundances in the former but high abundances in the latter. Like metacommunity D, 96.8% of samples (*n* = 153) in metacommunity H were carcinoma related. Both metacommunities D and H might represent a kind of widely existing metacommunity of high alpha diversity ecology in the colorectal cancer disease population (Fig. S5D and E). Metacommunity I, which mainly contained the CRC on-site tissue samples (*n* = 86; 61.9%), was characterized by the prevalence of *Fusobacterium* and *Parvimonas* (Fig. 6 and Fig. S5F) and showed substantially low chao1 metric alpha diversity (Fig. S5D).

### The cohort-to-cohort random forest model achieves better internal cohort classification.

**FIG 7:**
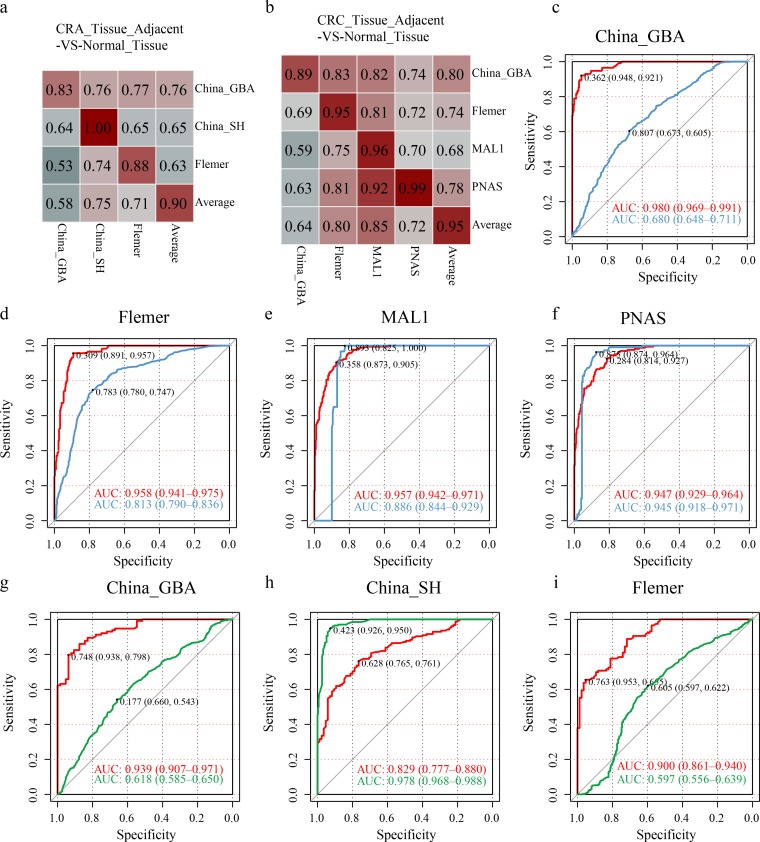
Cohort-to-cohort and leave-one-cohort-out random forest models. Classifier performances in the 10-fold cross-validation model within each cohort (along the diagonal, with the last number representing the average AUC) and the cohort-to-cohort training-testing models were measured by the AUC (off-diagonal, with the top *n*-1 row and column representing the training-testing data set, respectively [*n* is the cohort number]). The last row and column depict the average AUCs of the testing data sets to demonstrate the generalization ability to be predicted by multiple cohorts and predicting others. (a and b) Cohort-to-cohort performance for strategies CRA_Tissue_Adjacent-VS-Normal_Tissue and CRC_Tissue_Adjacent-VS-Normal_Tissue. (c to f) LOCO random forest models in training and testing carcinoma against normal samples using microbiota information of adjacent tissues. (g to i) LOCO random forest models in training and testing adenoma against normal samples using adjacent tissue microbiota information.

Although cohort information has been taken into consideration in pooling RF models and DEseq2 analyses, cohort-specific metacommunities were dominant, as shown by DMM analysis, regardless of disease status (Fig. 6). In order to characterize the reproducibility of our conclusion drawn by pooling all samples, we conducted random forest analysis with 10-fold cross-validation in each cohort and used the others as independent validation data sets in each strategy, here called the cohort-to-cohort random forest model (C2C RF model). Models utilizing adjacent tissues could discriminate adenoma and carcinoma from healthy tissues with AUC values of 0.90 and 0.95 on average in the training module (Fig. 7a and b).

However, weakness appeared in cross-cohort validations. Similar trends of high AUC values in training data sets but inferior AUC values in validation data sets could be observed for other strategies (Fig. S6A to F). Furthermore, when leaving one cohort out as the independent validation data set and training the rest in the CRC_Tissue_Adjacent-VS-Normal_Tissue strategy, better validation performance could be seen when the MAL1 and PNAS cohorts were left out for validation (Fig. 7c to f). In the CRA_Tissue_Adjacent-VS-Normal_Tissue strategy, when leaving cohorts China_GBA and Flemer out as validation data sets, the training model achieved AUC values of 0.939 and 0.90. When leaving the China_SH cohort out as the independent validation, we observed AUC values of 0.829 and 0.978 in the training and validation models, respectively (Fig. 7g to i).

10.1128/mSystems.00138-20.6 FIG S6 Performances of cohort-to-cohort random forests. (A to F) Cohort-to-cohort random forest models built for the CRA_Stools-VS-Normal_Stools, CRA_Tissue-VS-Normal_Tissue, CRA_Tissue-VS-CRA_Tissue_Adjacent, CRC_Stools-VS-Normal_Stools, CRC_Tissue-VS-Normal_Tissue, and CRC_Tissue-VS-CRC_Tissue_Adjacent strategies presented in the same schema as in Fig. 6. (G) Unique and shared features between cohort-to-cohort and pooling random forest models for eight strategies. Reproducible features for each strategy in the cohort-to-cohort model were chosen when appearing in at least 30% of training-testing pairs. Download FIG S6, PDF file, 0.9 MB. Copyright © 2020 Mo et al. 2020 Mo et al. This content is distributed under the terms of the Creative Commons Attribution 4.0 International license.

## DISCUSSION

Here, we confirmed that lesion-adjacent tissues were not as healthy as normal tissues, which was mentioned in previous small-sample-size research (7). Through PCoA, we identified distinct distributions of adjacent tissues compared with other sample groups. Second, we found a large number of overlapping features in strategies that discriminated lesion tissues and the lesion-adjacent tissues from normal tissues. Next, we found that the microbiome of lesion-adjacent tissues played an important role in supervised machine learning models that discriminated lesions from controls. We validated our hypothesis by calculating the network correlations between different sample groups, especially between lesion tissues and the surrounding adjacent ones. Finally, we showed that despite cohort heterogeneity, the microbial dysbiosis of lesion-adjacent tissues was validated to be a widespread phenomenon.

Compared to a single study, pooling of data sets from multiple studies enabled us to detect comprehensive alterations by strengthening the signal of relative abundance and reducing false positives with a comparable strict filtering standard to reject low-frequency taxa. When pooling samples from different cohorts, compared with the shotgun sequencing method using stool samples, both CRC and CRA models based on the 16S rRNA data set showed no distinct deficiency in the prediction (25). We characterized that lesion-adjacent and on-site lesion tissues could not be efficiently discriminated, as previously reported (6). As illustrated in our network analysis, the high microbial network correlation between lesion and surrounding lesion-adjacent tissues indicated that the microbial network configurations between them were highly similar (Fig. 5d to f). Besides network differences, a dynamic change of the relative abundance of a single feature in temporal order might help to identify driving tumorigenic factors in CRC development. Low relative abundances of *Ruminococcus*, *Blautia*, and *Dorea* were also reported in cancerous tissues (15). Interestingly, the dynamic fluctuation of the relative abundances of *Pseudomonas*, *Streptococcus*, *Porphyromonas*, and *Fusobacterium* in these sample groups, especially in adjacent tissues, might pave the way toward understanding their roles in tumorigenicity (Fig. 4).

Cohort heterogeneity is critical in affecting transcohort generalization. In pooling RF models, when some factors were included as binary features, we found that the China_GBA cohort factor ranked high in strategy S3: Adenoma_Tissue-VS-Normal_Tissue. This might be because 60% (52 out of 87) of adenoma tissue samples were from cohort China_GBA, whose data were from the 454 sequencing platform using the V1-V4 regions (see Fig. S4C in the supplemental material). In strategy S8, although the cohort China_GBA was inferior in discriminating CRC_Tissue_Adjacent from Normal_Tissue compared to cohorts MAL1, PNAS, and Flemer in the training model, it had a higher AUC score (0.80 on average) in independent testing cohorts. This suggested better training and testing generalizations (Fig. 7a and b). Particularly, when cohort China_GBA was left out as a validation data set, the poor performance in LOCO analysis in discriminating adenoma and carcinoma further confirmed cohort heterogeneity problems. Although limitations in machine learning performance were inevitable in the existing pipeline, especially in the adenoma stool-versus-control stool model (Fig. S6A to F), we still found comprehensive, reproducible features across cohorts in each strategy (Fig. S6G). For reproducible features, most of them were also identified in pooling random forest modules (Fig. S6G). All reproducible taxa in the eight strategies are summarized in Table S4.

10.1128/mSystems.00138-20.10 TABLE S4 Reproducible features across cohort-to-cohort random forest models. Shared_Features indicates the features shared between cohort-to-cohort and pooling random forest models, while cohort2cohort.Unique and Pooling.RF.Unique represent features that were unique in the cohort-to-cohort and pooling random forest models, respectively. Download Table S4, XLS file, 0.1 MB. Copyright © 2020 Mo et al. 2020 Mo et al. This content is distributed under the terms of the Creative Commons Attribution 4.0 International license.

Cohort heterogeneity was also observed regardless of disease status. High abundances of *Lactococcus* were observed in both the China_SH and China_SHTJ cohorts, suggesting that geography and ethnic groups were essential in shaping the specific microbiota community, as previously revealed (17), regardless of colorectal cancer disease status. We also observed that not each carcinoma sample was turbulent enough to be grouped into malignant groups, while some healthy samples were grouped with lesions. Some cohort-specific signatures harbored by metacommunity C might help to explain the high transcohort generalization testing results (Fig. 6h). Future research including a large number of samples of a specific ethnic cohort is encouraged to characterize cohort-specific tissue microbiome signatures and explain the driving factors shaping them.

Interestingly, since the microbiome component was distinctive in adjacent tissues, it might serve as an alternative for colorectal cancer screening, specifically for sigmoid cancer. The excellent performance in predicting cancer using the microbiota of surrounding adjacent cancerous tissues showed its potential for clinical application. It is challenging to obtain colorectal tissues of screening participants. However, according to previous studies sampling the mucosal microbiome (26–28), colonic lavage fluid, aspirated luminal contents, or the loose mucus layer could serve as a relatively accurate proxy in providing biopsy specimen microbiota compositions. Here, we examined this possibility by illustrating the following problems. First, there was no significant difference between strategies S7 and S8, indicating that the use of the adjacent tissue microbiome was sufficient for disease predictions. Second, Youden’s index maximizing the sum of sensitivities and specificities was applied to decide a threshold for the CRC_Tissue_Adjacent-VS-Normal_Tissue pooling RF model and achieved a sensitivity of 0.926 under a specificity of 0.858. For the CRA_Tissue_Adjacent-VS-Normal_Tissue model, the sensitivity was 0.726 under a specificity of 0.922 (Fig. 3b and c).

In the future application of the use of the gut microbiome to predict CRC/CRA, the mucosal microbiota might be an alternative and capable candidate for clinical application. Since some tissue samples used in our meta-analysis were not exactly mucosa but were biopsy specimens, lessons learned from currently available information remained incomplete. Further studies revealing the extent to which the distal mucosa microbiome represents the corresponding cancer-associated adjacent tissues are still needed. More investigations of the mucosa, especially the distal rectal mucosa microbiota, might help to develop a protocol that guides the sampling of the distal gastrointestinal tract mucosa noninvasively. For instance, mucosal-luminal interface (MLI) mucus (28), a mixture of the loose mucus layer sampled from the intestinal wall by washing off and aspirating, proved to harbor a biomass highly similar to that of biopsy specimens (29, 30) and might serve as a replacement for biopsy specimens. Other methods like colon swap could be used as an in-house device if qualified to capture crucial mucosal microbiome signatures. Furthermore, a new tool designed to scrape the colon mucosa by clinician rectum examination could also serve as an alternative instrument. In this way, carcinomas and adenomas located on the sigmoid colon might be detected with high sensitivities and specificities based on the knowledge that the lesion-adjacent mucosa microbiota plays a crucial role in making prediction models.

In our further research, some other improvements might help to obtain better performance. First, although the feature-based method could effectively utilize microbiome information in disease classifications, unlike *de novo* OTU picking protocols that took advantage of each filtered read, some reads were rejected by either Trimmomatic (31) or Kraken2 (20) in our pipeline, resulting in the omission of some unknown taxa which might be potential markers in specific case-control models. Second, the sample read numbers differed from cohort to cohort in magnitude, which made it difficult to perform rarefaction. To keep more details of read information, after checking that the feature number would reach a plateau in each cohort, read numbers were normalized to obtain relative feature abundances without rarefaction for downstream analyses. In the planned compatibility progress, the concordant sample preparation pipeline might help identify vital functional elements in different types of samples unfailingly.

Finally, we would like to integrate other possible confounding factors, like age, gender, body mass index (BMI), tumor location, methods for obtaining biopsy specimens, and cancer status, in our future studies. For unity consideration, mucosa and biopsy specimens were combined and termed “tissue” in our analyses. Moreover, we used a filtering pipeline to remove bacterial taxa existing in fewer than 10% of samples after read normalization. While minimizing the false-positive discovery rate, this might have led to the missing of some rare but interesting taxa.

In conclusion, we identified significant dysbiosis in both lesions and lesion-adjacent tissues compared with healthy colon tissues. We also found that judged from the microbiome component perspective, lesion-adjacent tissues should not be regarded as healthy colon tissues. This research provided new perspectives for further research in revealing the role of the microbiome in tumorigenesis along with the development of colorectal tumors.

## MATERIALS AND METHODS

### Data set collection and sequencing data preprocessing.

The 16 data sets were labeled as Zeller (5), Flemer (32), Burns (33), Baxter (10), China_GBA (China Great Bay Area) (23), MAL2 (34), MAL1 (34), Zackular (9), Kostic (35), PNAS (36), Brazil (19), China_KM (China, Kunming) (37), China_SH (38), China_QD (China, Qingdao) (39), and China_SHTJ (40). Raw sequence data and metadata were retrieved from the NCBI or from the authors directly. All sequences were trimmed by using Trimmomatic (31) as described in previous research (41). For sequences generated by the Illumina platform, Illumina-specific adapters were removed using default parameters. Samples were excluded if metadata were not available. Samples were grouped before downstream analyses. First, stool samples derived from healthy controls and adenoma and CRC patients were grouped as Normal_Stools, CRA_Stools, and CRC_Stools, respectively. Second, tissue samples from healthy control and adenoma and carcinoma disease sites were grouped as Normal_Tissue, CRA_Tissue, and CRC_Tissue, respectively. Third, we grouped the surrounding tissue samples (usually 5 to 10 cm away from the lesion) that were adjacent to the cancerous sites as CRA_Tissue_Adjacent and CRC_Tissue_Adjacent for adenoma and carcinoma patients.

### Sequence classification, taxonomy determination, and feature relative abundance.

The Kraken2 (20) algorithm was applied to classify each high-throughput sequence read directly against the GreenGenes database and return their taxa. Each sample was processed independently to gain a mataphlan2 (42) format report of microbiome compositions, with features ranging from kingdom to species. The Kraken2 report was filtered under the criterion that each feature must exist in at least 10% of all samples.

### Beta diversity and principal-coordinate analyses.

Feature relative abundance-based information was subjected to calculation of the beta diversity using Bray-Curtis dissimilarity metrics via a module implemented in Qiime (43) software. Subsequently, we performed principal-coordinate analysis (PCoA) based on our Bray-Curtis dissimilarity matrix.

### Use of the random forest machine learning model to discriminate sample groups.

All the random forest models were built using the supervised_learning.py command in Qiime software (version 1.9.1) (43). This script was called by the randomForest R package (version 4.6-14) and was used to perform random forest analysis with default parameters using inner 10-fold cross-validation to avoid overfitting. All returned feature importance scores were characterized using MDIA to present the importance of features in model classifications. In the optimal feature number decision procedure, all features were included to obtain the importance of each feature, from which they were sorted. Next, the above-mentioned models were repeated, with previously ranked features added one by one, starting from the most important one. The optimized model that made the AUC value reach a plateau or peak was selected. Finally, all the resulting probabilities served as the input for the pROC R packages to compute the AUC values and draw the ROC (receiver operating characteristic). A similar feature selection procedure was applied to cohort-to-cohort and leave-one-cohort-out (LOCO) RF models, in which we added an additional prediction function to use independent data sets for validation to evaluate the generalization of trained models and gained each tested sample’s probabilities of being assigned to different groups. In each circle of added features, the model that maximized the sum of training and validation AUCs was chosen, and the corresponding features were determined as potential markers for downstream analyses.

### Feature cross talk.

The importance of each feature was represented by ranking according to the MDIA in the pooling RF model using 10-fold cross-validation. Features ranking in the top 25 in the corresponding strategy were selected and separated into family, genus, and species taxonomy groups.

### Correlation network inference.

The filtered features (genus-level and species-level features that existed in at least 20% of 2,099 samples) were subjected to the SparCC (22) algorithm to calculate the taxon-taxon correlation coefficient matrix for each group of samples using default parameters. Correlation coefficient matrices of each group of samples were sorted in the same taxonomy order and were applied to compare network similarities.

### Cohort-to-cohort reproducible features.

For cohort-to-cohort RF models in each strategy (*n* = 8), each cohort served as a training data set and was tested using others, resulting in *n* × (*n* − 1) training-testing pairs, as demonstrated, with *n* representing the cohort numbers. For the reason that some training-testing pairs reached optimal status by maximizing the sum of the discovery AUC and the validation AUC using only a few features, features prevailing in at least 30% of pairs were regarded as highly reproducible.

### Determination of enterotype for tissue samples.

Relative abundance-transformed counts of 657 filtered features were subjected to the DMM algorithm (44) to identify groups of metacommunities harboring similar microbial configurations using Mothur (45) software with default parameters for tissue samples. Nine metacommunities were obtained based on the BIC approximation. Subsequently, only genus and species features presenting significantly different profiles in both DEseq2 and pooling RF models in 8 strategies are shown in a heat map. Different enrichment patterns of the microbiota were hierarchically clustered using the Pearson correlation, as presented in rows. Alpha diversity regarding this part was performed based on relative abundance-transformed feature counts.

### Statistical analysis.

The Mann-Whitney test was applied to compute the paired-sample difference and significance, and the Kruskal-Wallis rank sum test was used for multiple samples. DEseq2 (46) was chosen to conduct differential feature abundance analyses with cohort information added to adjust the batch effect. Log_2_-transformed fold changes and adjusted *P* values served as factors for downstream screening. The ade4 (47) R package was used to compute 95% of the inertia in the PCoA modules for each group. The 95% confidence interval of the ROC was calculated with 2,000 stratified bootstrap replicates, and DeLong’s test was conducted for two ROC curves using the pROC R package (48). The Mantel test was applied to compute the similarity and significance between matrices using the two-sided method implemented in the ade4 package (47).
