# Supplementary figures and images for: Meta-analysis of 16S rRNA Microbial Data Identified Distinctive and Predictive Microbiota Dysbiosis in Colorectal Carcinoma Adjacent Tissue
Source: mSystems. 2020 Apr 14;5(2):e00138-20. doi: 10.1128/mSystems.00138-20 (PMC7159898; doi:10.1128/mSystems.00138-20)

# Reads profiles

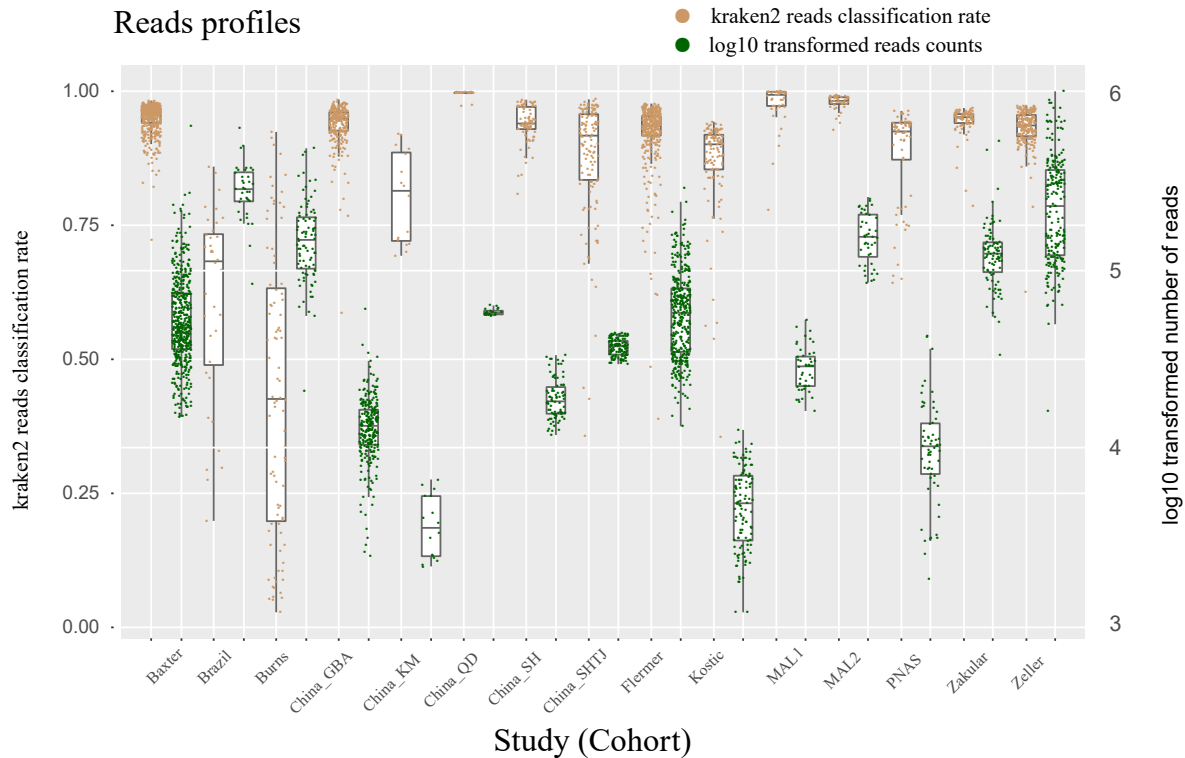

Supplement: FIG S1 [file mSystems.00138-20-sf001.pdf]

A

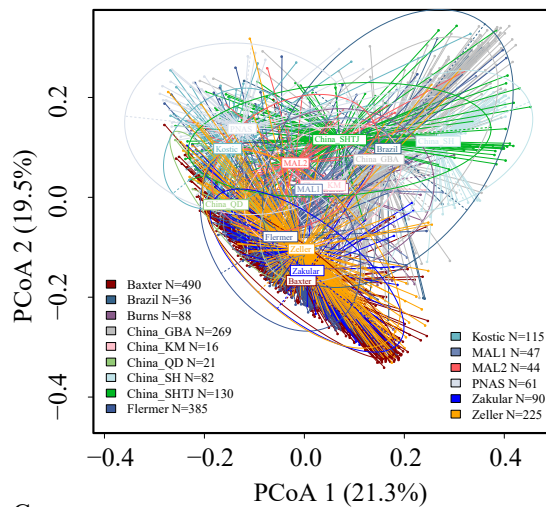

B

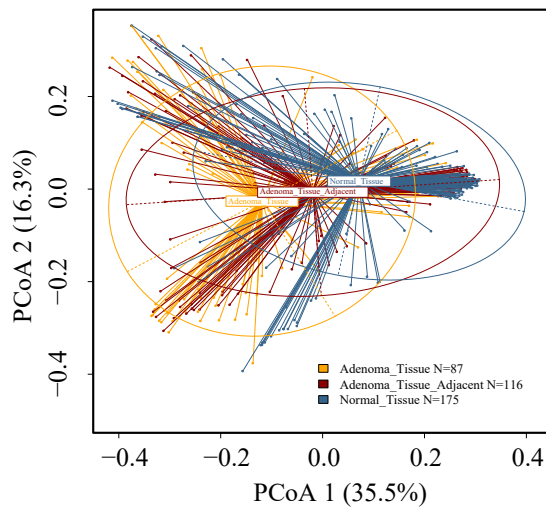

C

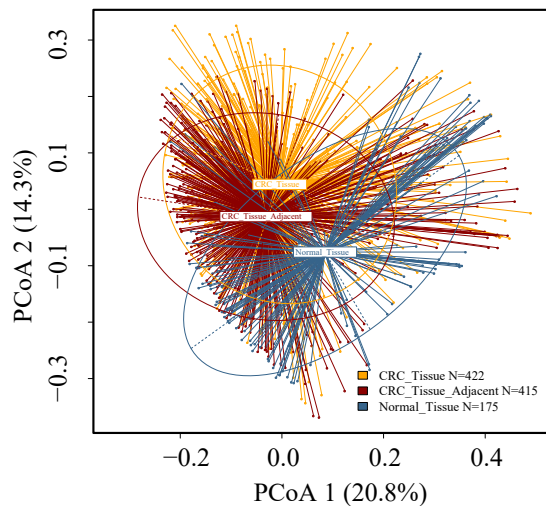

D

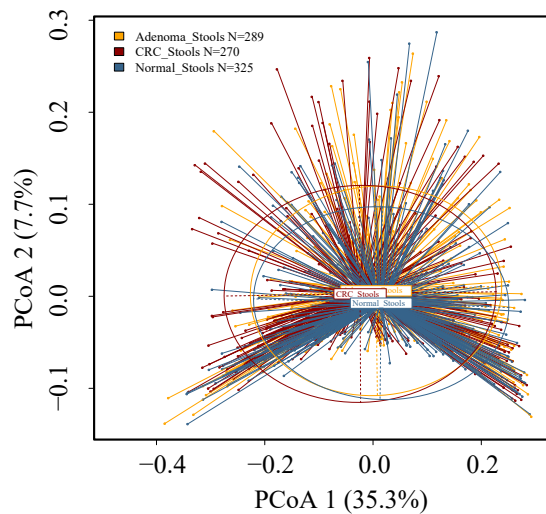

Supplement: FIG S2 [file mSystems.00138-20-sf002.pdf]

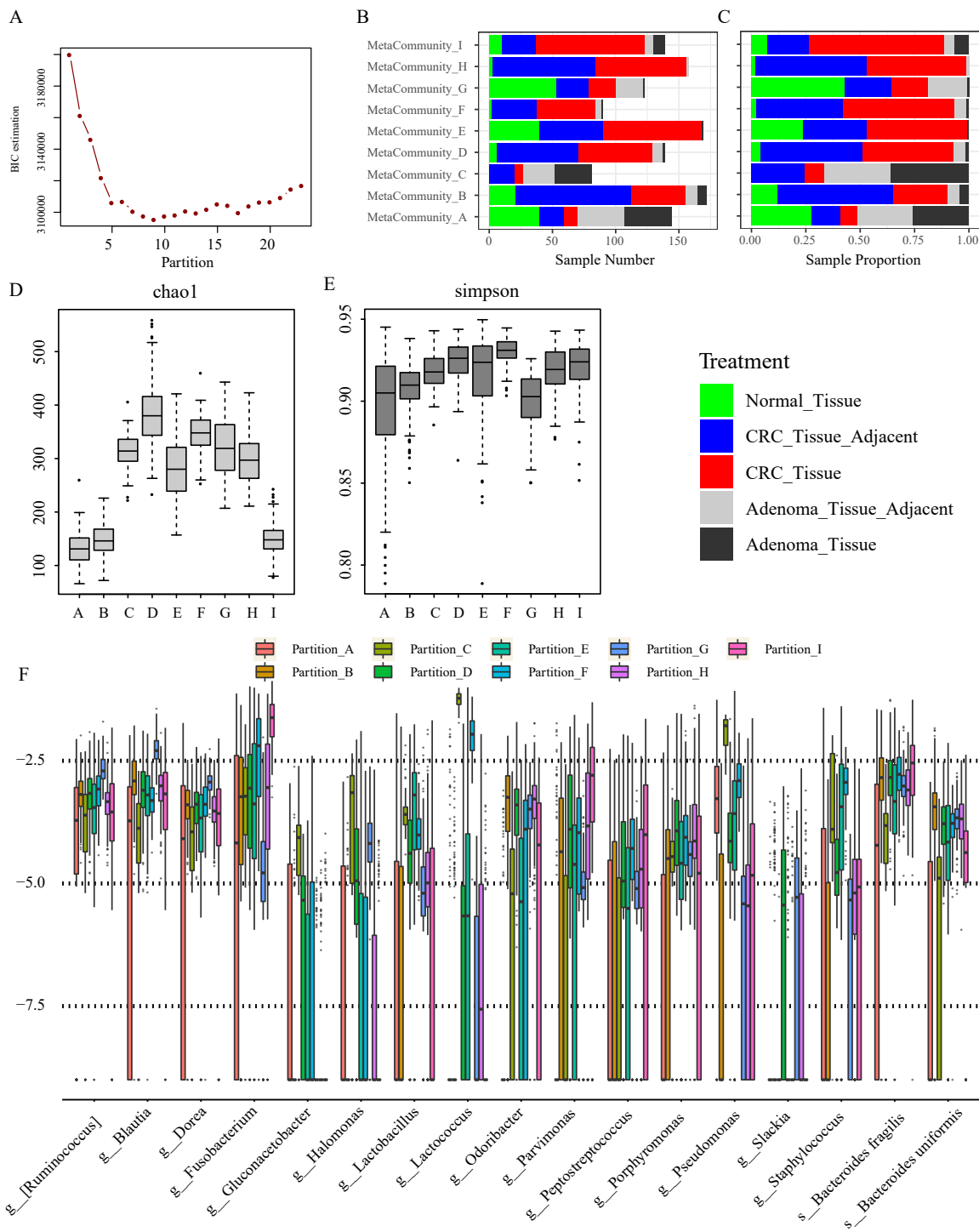

Supplement: FIG S5 [file mSystems.00138-20-sf005.pdf]
