# Supplementary material for: Meta-analysis of 16S rRNA Microbial Data Identified Distinctive and Predictive Microbiota Dysbiosis in Colorectal Carcinoma Adjacent Tissue
Source: mSystems. 2020 Apr 14;5(2):e00138-20. doi: 10.1128/mSystems.00138-20 (PMC7159898; doi:10.1128/mSystems.00138-20)
Supplement: FIG S3 [file mSystems.00138-20-sf003.pdf]

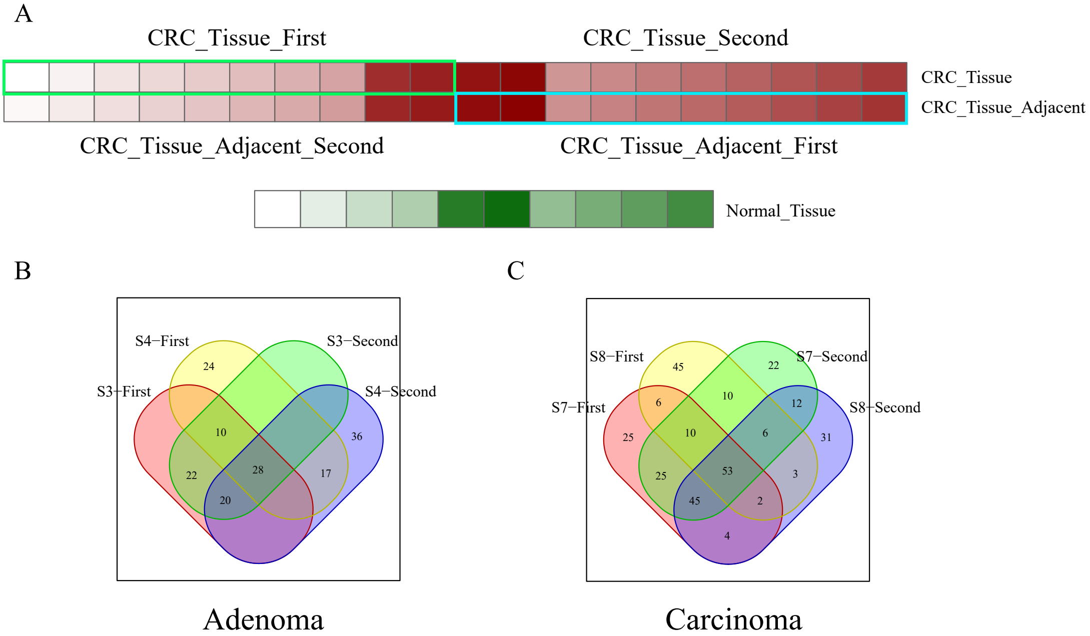

S3-First: CRA_Tissue_First-VS-Normal_Tissue
S4-First: CRA_Tissue_Adjacent_First-VS-Normal_Tissue
S3-Second: CRA_Tissue_Second-VS-Normal_Tissue
S4-Second: CRA_Tissue_Adjacent_Second-VS-Normal_Tissue

S7-First: CRC_Tissue_First-VS-Normal_Tissue
S8-First: CRC_Tissue_Adjacent_First-VS-Normal_Tissue
S7-Second: CRC_Tissue_Second-VS-Normal_Tissue
S8-Second: CRC_Tissue_Adjacent_Second-VS-Normal_Tissue
