# Supplementary material for: Meta-analysis of 16S rRNA Microbial Data Identified Distinctive and Predictive Microbiota Dysbiosis in Colorectal Carcinoma Adjacent Tissue
Source: mSystems. 2020 Apr 14;5(2):e00138-20. doi: 10.1128/mSystems.00138-20 (PMC7159898; doi:10.1128/mSystems.00138-20)
Supplement: FIG S4 [file mSystems.00138-20-sf004.pdf]

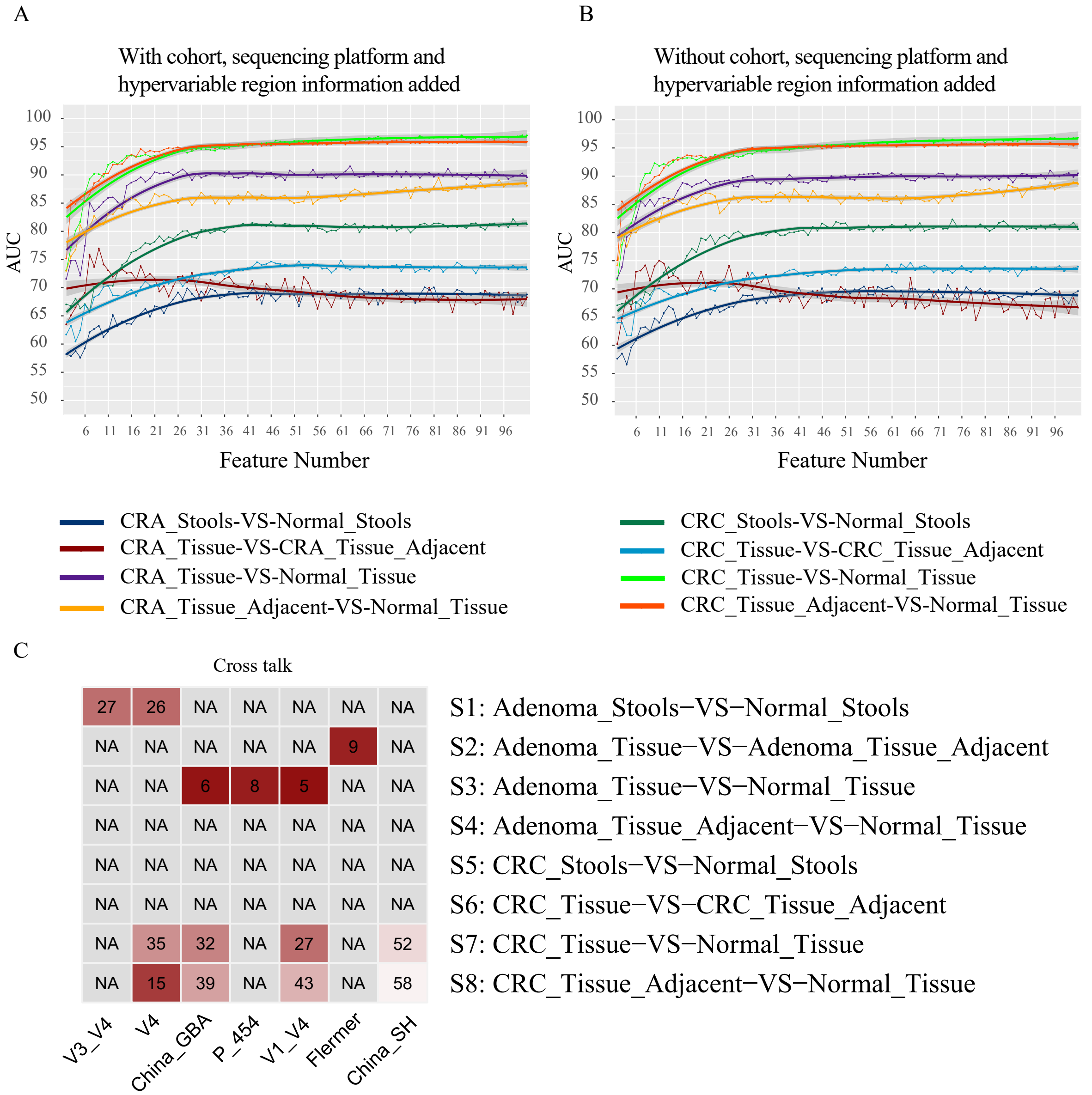
A
With cohort, sequencing platform and hypervariable region information added
B
Without cohort, sequencing platform and hypervariable region information added
AUC
100
95
90
85
80
75
70
65
60
55
50
6 11 16 21 26 31 36 41 46 51 56 61 66 71 76 81 86 91 96
Feature Number
CRA_Stools-VS-Normal_Stools
CRA_Tissue-VS-CRA_Tissue_Adjacent
CRA_Tissue-VS-Normal_Tissue
CRA_Tissue_Adjacent-VS-Normal_Tissue
CRC_Stools-VS-Normal_Stools
CRC_Tissue-VS-CRC_Tissue_Adjacent
CRC_Tissue-VS-Normal_Tissue
CRC_Tissue_Adjacent-VS-Normal_Tissue
C
Cross talk
27 26 NA NA NA NA NA
NA NA NA NA NA 9 NA
NA NA 6 8 5 NA NA
NA NA NA NA NA NA NA
NA NA NA NA NA NA NA
NA NA NA NA NA NA NA
NA 35 32 NA 27 NA 52
NA 15 39 NA 43 NA 58
V3_V4
V4
China_GBA
P_454
V1_V4
Flermer
China_SH
S1: Adenoma_Stools−VS−Normal_Stools
S2: Adenoma_Tissue−VS−Adenoma_Tissue_Adjacent
S3: Adenoma_Tissue−VS−Normal_Tissue
S4: Adenoma_Tissue_Adjacent−VS−Normal_Tissue
S5: CRC_Stools−VS−Normal_Stools
S6: CRC_Tissue−VS−CRC_Tissue_Adjacent
S7: CRC_Tissue−VS−Normal_Tissue
S8: CRC_Tissue_Adjacent−VS−Normal_Tissue
