# Supplementary material for: Meta-analysis of 16S rRNA Microbial Data Identified Distinctive and Predictive Microbiota Dysbiosis in Colorectal Carcinoma Adjacent Tissue
Source: mSystems. 2020 Apr 14;5(2):e00138-20. doi: 10.1128/mSystems.00138-20 (PMC7159898; doi:10.1128/mSystems.00138-20)
Supplement: FIG S6 [file mSystems.00138-20-sf006.pdf]

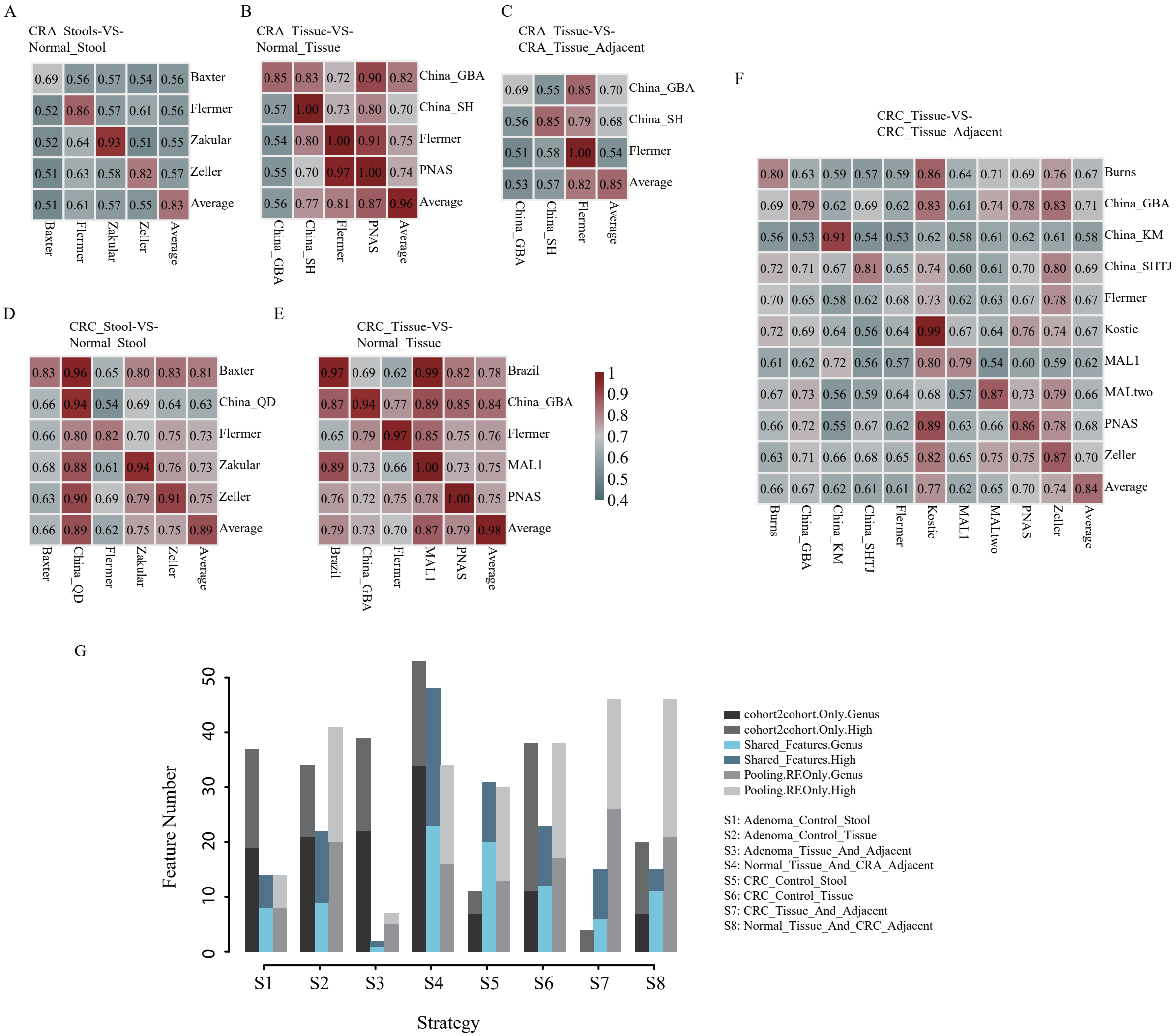
A
CRA_Stools-VS-Normal_Stool
0.69 0.56 0.57 0.54 0.56 Baxter
0.52 0.86 0.57 0.61 0.56 Flermer
0.52 0.64 0.93 0.51 0.55 Zakular
0.51 0.63 0.58 0.82 0.57 Zeller
0.51 0.61 0.57 0.55 0.83 Average
Baxter Flermer Zakular Zeller Average
B
CRA_Tissue-VS-Normal_Tissue
0.85 0.83 0.72 0.90 0.82 China_GBA
0.57 1.00 0.73 0.80 0.70 China_SH
0.54 0.80 1.00 0.91 0.75 Flermer
0.55 0.70 0.97 1.00 0.74 PNAS
0.56 0.77 0.81 0.87 0.96 Average
China_GBA China_SH Flermer PNAS Average
C
CRA_Tissue-VS-CRA_Tissue_Adjacent
0.69 0.55 0.85 0.70 China_GBA
0.56 0.85 0.79 0.68 China_SH
0.51 0.58 1.00 0.54 Flermer
0.53 0.57 0.82 0.85 Average
China_GBA China_SH Flermer Average
D
CRC_Stool-VS-Normal_Stool
0.83 0.96 0.65 0.80 0.83 0.81 Baxter
0.66 0.94 0.54 0.69 0.64 0.63 China_QD
0.66 0.80 0.82 0.70 0.75 0.73 Flermer
0.68 0.88 0.61 0.94 0.76 0.73 Zakular
0.63 0.90 0.69 0.79 0.91 0.75 Zeller
0.66 0.89 0.62 0.75 0.75 0.89 Average
Baxter China_QD Flermer Zakular Zeller Average
E
CRC_Tissue-VS-Normal_Tissue
0.97 0.69 0.62 0.99 0.82 0.78 Brazil
0.87 0.94 0.77 0.89 0.85 0.84 China_GBA
0.65 0.79 0.97 0.85 0.75 0.76 Flermer
0.89 0.73 0.66 1.00 0.73 0.75 MAL1
0.76 0.72 0.75 0.78 1.00 0.75 PNAS
0.79 0.73 0.70 0.87 0.79 0.98 Average
Brazil China_GBA Flermer MAL1 PNAS Average
1 0.9 0.8 0.7 0.6 0.5 0.4
F
CRC_Tissue-VS-CRC_Tissue_Adjacent
0.80 0.63 0.59 0.57 0.59 0.86 0.64 0.71 0.69 0.76 0.67 Burns
0.69 0.79 0.62 0.69 0.62 0.83 0.61 0.74 0.78 0.83 0.71 China_GBA
0.56 0.53 0.91 0.54 0.53 0.62 0.58 0.61 0.62 0.61 0.58 China_KM
0.72 0.71 0.67 0.81 0.65 0.74 0.60 0.61 0.70 0.80 0.69 China_SHTJ
0.70 0.65 0.58 0.62 0.68 0.73 0.62 0.63 0.67 0.78 0.67 Flermer
0.72 0.69 0.64 0.56 0.64 0.99 0.67 0.64 0.76 0.74 0.67 Kostic
0.61 0.62 0.72 0.56 0.57 0.80 0.79 0.54 0.60 0.59 0.62 MAL1
0.67 0.73 0.56 0.59 0.64 0.68 0.57 0.87 0.73 0.79 0.66 MALtwo
0.66 0.72 0.55 0.67 0.62 0.89 0.63 0.66 0.86 0.78 0.68 PNAS
0.63 0.71 0.66 0.68 0.65 0.82 0.65 0.75 0.75 0.87 0.70 Zeller
0.66 0.67 0.62 0.61 0.61 0.77 0.62 0.65 0.70 0.74 0.84 Average
Burns China_GBA China_KM China_SHTJ Flermer Kostic MAL1 MALtwo PNAS Zeller Average
G
Feature Number
0 10 20 30 40 50
S1 S2 S3 S4 S5 S6 S7 S8
Strategy
cohort2cohort.Only.Genus
cohort2cohort.Only.High
Shared_Features.Genus
Shared_Features.High
Pooling.RF.Only.Genus
Pooling.RF.Only.High
S1: Adenoma_Control_Stool
S2: Adenoma_Control_Tissue
S3: Adenoma_Tissue_And_Adjacent
S4: Normal_Tissue_And_CRA_Adjacent
S5: CRC_Control_Stool
S6: CRC_Control_Tissue
S7: CRC_Tissue_And_Adjacent
S8: Normal_Tissue_And_CRC_Adjacent
